# The risk of new‐onset cancer associated with HFE C282Y and H63D mutations: evidence from 87,028 participants

**DOI:** 10.1111/jcmm.12764

**Published:** 2016-02-19

**Authors:** Yang‐Fan Lv, Xian Chang, Rui‐Xi Hua, Guang‐Ning Yan, Gang Meng, Xiao‐Yu Liao, Xi Zhang, Qiao‐Nan Guo

**Affiliations:** ^1^ Department of PathologyXinqiao HospitalThe Third Military Medical UniversityChongqingChina; ^2^ Department of OrthopedicsXinqiao HospitalThe Third Military Medical UniversityChongqingChina; ^3^ Department of OncologyThe First Affiliated Hospital of Sun Yat‐sen UniversityGuangzhouGuangdongChina; ^4^ Department of EndocrinologyXinqiao HospitalThird Military Medical UniversityChongqingChina

**Keywords:** hereditary haemochromatosis, HFE, mutation, C282Y, H63D, meta‐analysis, cancer

## Abstract

To investigate the association between mutation of HFE (the principal pathogenic gene in hereditary haemochromatosis) and risk of cancer, we conducted a meta‐analysis of all available case–control or cohort studies relating to two missense mutations, C282Y and H63D mutations. Eligible studies were identified by searching databases including PubMed, Embase and the ISI Web of Knowledge. Overall and subgroup analyses were performed and odds ratios (ORs) combined with 95% confidence intervals (CIs) were applied to evaluate the association between C282Y mutation, H63D mutation and cancer risk. Sensitivity and cumulative analyses were used to evaluate the stability of the results. A total of 36 eligible studies were included, comprising 13,680 cases and 73,348 controls. C282Y was significantly associated with elevated cancer risk in a recessive genetic model (OR: 1.991, 95% CI: 1.448–2.737). On subgroup analysis stratified by cancer type, statistically significantly increased cancer risks were found for breast cancer, colorectal cancer and hepatocellular carcinoma in a recessive model. When stratified by territory, a significantly increased risk of cancer was found in Oceanic populations in a recessive model and in Asian populations in an allele model and dominant model. H63D mutation did not significantly increase overall cancer risk in any genetic model. However, when, stratified by territory, an increased cancer risk was found in the Asian population in an allele and dominant. C282Y but not H63D mutation was related to elevated cancer risk. Further large‐scale studies considering gene–environment interactions and functional research should be conducted to further investigate this association.

## Introduction

Hereditary haemochromatosis is an autosomal recessive disease, the principal pathogenic gene of which is HFE 1, 2. The condition is characterized by a disorder of intestinal iron absorption that causes progressive accumulation of iron in organs including the liver, heart and pancreas, leading to their dysfunction 3. An important pathogenic mechanism may the catalytic activity of iron in the formation of hydroxyl radicals. Iron may also suppress host defence cell activity and promote cancer cell proliferation. It is increasingly reported that two mutations in HFE – C282Y (rs1800562G>A) and H63D (rs1799945 C>G) – are associated with an increased risk of cancers, including hepatocellular 4, 5, breast 6, colorectal 7 and prostate cancer 8, as well as others 9, 10, 11, 12. However, some other studies have shown no association between haemochromatosis genotype and neoplasia 13, 14, 15, 16. This controversy warrants further studies.

In 1996, C282Y and H63D were shown to be related to altered iron status 17. The damage caused by iron overload is associated with oxidative stress, and several studies have demonstrated iron overload to be correlated with carcinogenesis 18. A number of studies have investigated the association between C282Y and H63D and an increased cancer risk. However, the studies have been underpowered and the findings have proved somewhat controversial. For, a meta‐analysis in 2010 by Jin *et al*. 4 found a significant association between C282Y and H63D and hepatocellular carcinoma. However, they included a cross‐sectional 19. Moreover, there are now a number of other studies reported 14, 20, 21, 22, 23. In 2013, Chen *et al*. reported a significant association between C282Y and colorectal cancer. They only used a recessive model and classified all those from the United States as Caucasians 24. In the same year, Liu *et al*. reported similar findings. They classified those from the United States and Brazil as being Europeans 25.

In our study, we have employed cumulative analysis, which has not been previously used. To the best of our knowledge, this is the most comprehensive meta‐analysis of C282Y and H63D HFE mutations and the risk of cancer. We included 36 studies, comprising 13,680 cases and 73,348 controls. The malignancies studied were principally hepatocellular, breast, colorectal and prostate carcinomas and acute leukaemia.

## Materials and methods

### Study identification and selection criteria

We searched PubMed, Embase, the ISI Web of Knowledge, the Chinese Biomedical database and the China National Knowledge Infrastructure to identify relevant studies, from which only case–control and cohort studies published between December 1995 and May 2014 were selected. The terms ‘Case–Control Studies or Cohort Studies’, ‘Neoplasms or Carcinoma’, ‘Alleles or SNP or Genetic Variation or Mutation or Polymorphism’ and ‘Haemochromatosis or HFE or C282Y or H63D’ were combined. The reference lists and related articles were also scrutinized to identify additional studies.

This study was performed according to the Newcastle–Ottawa Scale (NOS) for meta‐analysis of observational studies 26. The NOS uses a star system (range, 0–9 stars) for evaluating the quality of such studies, allowing a mean value of included studies to be calculated. Articles were selected if they met all of the following criteria: (*i*) the study was a case–control study or cohort study concerning the association between the haemochromatosis genotype C282Y or H63D and risk of cancer; (*ii*) the articles provided data on the distribution of the alleles, the size of the sample and number of controls, the exact number of each genotype or other information to aid the calculations; (*iii*) neoplasms were diagnosed by histopathological biopsy and the controls were free from cancer; and (*iv*) the publication language was English or Chinese. The control group included in our study were hospitalized controls or randomly selected from a pool of eligible participants matched to the index case by age, sex and township of residence.

### Data extraction

Two authors (Yang‐fan Lv and Xian Chang) extracted information independently from the selected studies. The results were compared and collated, and contradictions were resolved by discussion or by consultation with the corresponding author of the study in question. The data extracted were: first author name; title of article; publication year; country where study was performed; territory of participants; HFE mutation type; precise size of case and control groups; and distribution of genotypes in both case and control groups.

### Statistical methods

The control groups of all of the included articles were tested for Hardy–Weinberg equilibrium 27. The strength of the association between HFE genotypes and cancer risk was measured by the odds ratio (OR) with 95% confidence intervals (CIs). *P*_OR_ < 0.05 was regarded as statistically significant. Subgroup meta‐analyses were performed according to cancer type and territory for both C282Y and H63D, independently. The chi‐squared test and *I* ^2^ statistic were used to evaluate heterogeneity 28. *P*‐values less than 0.10 indicated heterogeneity among studies and a random‐effects model was used to estimate the pooled OR. Otherwise, a fixed‐effects model was used. Sensitivity analysis was performed to evaluate the impact of the studies and the stability of the results. To investigate the dynamic trend of the association between HFE mutation and cancer risk, cumulative analysis was performed according to year of publication and sample size 29. Furthermore, Begg's test 30 and Egger's test 31 were performed to assess the publication bias of the literature 30, 32. *P* < 0.05 was considered statistically significant. All statistical tests were performed with STATA 12.0 software 31. Finally, to adjust for multiple comparisons, the Bonferroni method were applied (see Tables S1 and S2).

## Results

### Eligible studies

**Figure 1:**
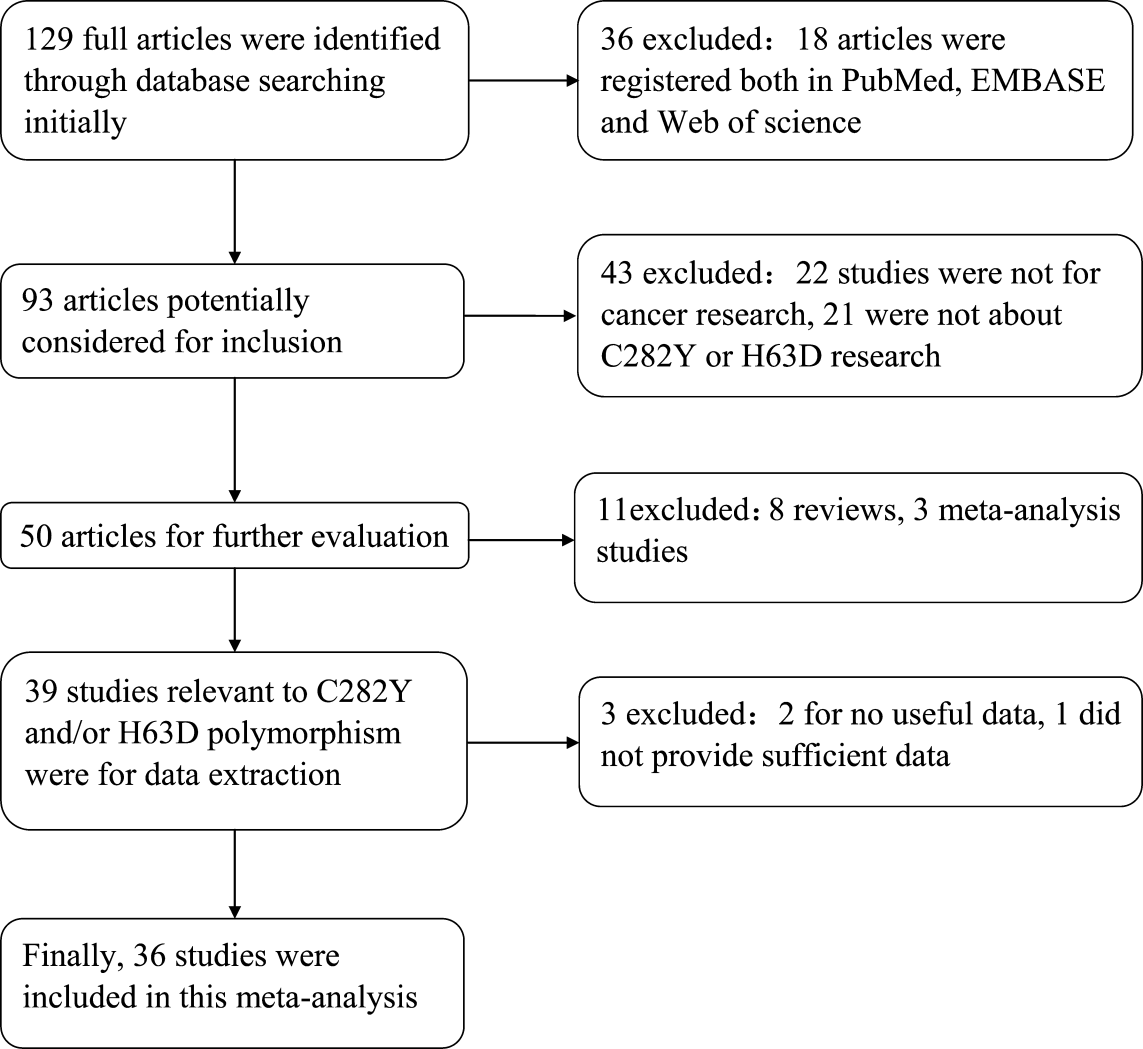
Flow chart for inclusion of studies.

One hundred and twenty‐nine studies were found concerning the association between HFE mutation and cancer risk. Following a review of all articles according to the criteria (shown in Fig. 1), 36 eligible studies were included in our pooled analysis. Among these, 33 7, 8, 10, 12, 13, 14, 15, 16, 20, 21, 22, 23, 33, 34, 35, 36, 37, 38, 39, 40, 41, 42, 43, 44, 45, 46, 47, 48, 49, 50, 51, 52, 53 were concerned with C282Y, 30 6, 7, 8, 9, 10, 12, 13, 14, 16, 20, 22, 23, 38, 39, 40, 41, 42, 43, 44, 45, 46, 47, 48, 49, 50, 51, 52, 53 with H63D and 27 7, 8, 10, 12, 13, 14, 15, 16, 20, 22, 23, 38, 39, 40, 41, 42, 43, 44, 45, 46, 47, 48, 49, 50, 51, 52, 53 with both C282Y and H63D. The principal characteristics of the studies concerning C282Y and H63D are listed in Tables 1 and 2. It should be noted that one study 56 was excluded because it did not provide sufficient data of the distribution of genotypes in both case and control groups.

**Table 1:** Main characteristics of all case–control or cohort studies included in H63D and cancer risk

First author	Year	Study design	Country	Territory	Cancer type	Sample size	Case			Control		
						Case/control	CC	CW	WW	CC	CW	WW
Beckman	1999	Case–control	Sweden	European	Breast	165/294	1	25	139	4	35	255
Altes	1999	Case–control	France	European	Colorectal	73/76	0	5	68	0	6	70
Beckman	1999	Case–control	Sweden	European	Colorectal	173/294	2	21	150	4	35	255
Gimferrer	1999	Case–control	Spain	European	AL	36/106	0	3	33	0	6	100
Racchi	1999	Case–control	Italy	European	Hepatocellular	15/130	0	3	12	0	11	119
Beckman	2000	Case–control	Sweden	European	Hepatocellular	54/294	1	10	43	1	38	255
Parkkila	2001	Case–control	Finland	European	AL	18/102	0	0	18	0	10	92
Fargion	2001	Case–control	Italy	European	Hepatocellular	81/128	0	7	74	0	2	126
Campo S	2001	Case–control	Italy	European	Hepatocellular	23/304	0	0	23	0	1	303
Lauret	2002	Case–control	Spain	European	Hepatocellular	77/359	0	12	65	0	22	337
Boige	2003	Case–control	France	European	Hepatocellular	133/100	0	7	126	1	6	93
Cauza	2003	Case–control	Australia	Oceanican	Hepatocellular	162/671	5	18	139	5	63	603
Shaheen	2003	Case–control	United States	North American	Colorectal	475/833	0	44	431	3	68	762
Hellerbrand	2003	Case–control	Germany	European	Hepatocellular	137/233	0	17	120	0	10	223
van der	2003	Case–control	Netherlands	European	Colorectal	191/573	0	16	175	3	38	532
Kallianpur	2004	Cohort	United States	North American	Breast	41/129	5	10	26	7	15	107
Abraham	2005	Case–control	Germany	European	Breast	566/649	2	59	505	1	71	577
McGlynn	2005	Case–control	United States	North American	Colorectal	635/650	5	70	560	3	76	571
Robinson	2005	Case–control	United Kingdom	European	Colorectal	327/322	2	50	275	4	39	279
Shi	2005	Case–control	China	Asian	Hepatocellular	56/60	6	3	47	0	1	59
Festa	2005	Case–control	Sweden	European	Basal cell	241/259	2	17	222	1	22	236
Syrjakoski	2006	Cohort	Finland	European	Prostatic	843/480	9	55	779	3	45	432
Syrjakoski	2006	Cohort	Finland	European	Breast	116/480	1	5	110	3	45	432
Cardoso	2006	Case–control	Portugal	European	Cervical	150/91	0	14	136	1	5	85
Kondrashova	2006	Case–control	Russia	European	Breast	100/260	0	2	98	0	17	243
Ropero	2007	Case–control	Spain	European	Hepatocellular	196/181	1	12	183	0	23	158
Yonal	2007	Case–control	Turkey	Asian	Hepatocellular	19/251	0	0	19	2	2	247
Hucl	2007	Case–control	Germany	European	Pancreatic	117/428	1	7	109	1	30	397
Nahon	2008	Cohort	France	European	Hepatocellular	103/198	0	12	91	0	18	180
Ezzikouri	2008	Case–control	France	European	Hepatocellular	96/222	0	2	94	0	3	219
Shi	2009	Case–control	Australia	Oceanica	Colorectal	85/3079	0	16	69	16	424	2639
Shi	2009	Case–control	Polish	European	Colorectal	75/1622	0	1	74	2	123	1497
Osborne	2010	Cohort	Australia	Oceanican	Colorectal	620/28,414	10	80	530	193	3882	24,339
Osborne	2010	Cohort	Australia	Oceanican	Breast	664/16,399	9	90	565	90	2263	14,046
Gannon	2011	Cohort	Canada	North American	Ovarian	354/80	2	32	320	0	2	78
Ekblom	2012	Cohort	Sweden	European	Colorectal	211/400	2	27	182	1	47	352
Rodriguez‐Lopez	2013	Case–control	Spain	European	AL	59/173	0	2	57	0	16	157
Total						7487/59,324						

C indicates C282Y mutant and W indicates wild‐type respectively, AL indicates acute leukaemia.

**Table 2:** Main characteristics of all case–control or cohort studies included in H63D and cancer risk

First author	Year	Study design	Country	Territory	Cancer type	Sample size	Case			Control		
						Case/control	HH	HW	WW	HH	HW	WW
Racci	1999	Case–control	Italy	European	Hepatocellular	12/130	0	3	9	3	42	85
Gimferrer	1999	Case–control	Spain	European	AL	36/106	2	11	23	2	28	76
Altes	1999	Case–control	France	European	Colorectal	110/100	6	36	68	2	28	70
Beckman	2000	Case–control	Sweden	European	Hepatocellular	54/294	0	17	37	6	59	229
Campo S	2001	Case–control	Italy	European	Hepatocellular	23/304	1	6	16	12	90	202
Martinez	2001	Case–control	Italy	European	Gliomas	174/144	6	56	112	2	32	110
Lauret	2002	Case–control	Spain	European	Hepatocellular	77/359	0	25	52	33	92	234
Boige	2003	Case–control	France	European	Hepatocellular	133/100	0	41	92	1	40	59
Cauza	2003	Case–control	Australia	Oceanican	Hepatocellular	162/671	3	31	128	9	133	529
Shaheen	2003	Case–control	United States	North American	Colorectal	475/833	10	88	377	12	135	686
Hellerbrand	2003	Case–control	Germany	European	Hepatocellular	137/233	2	27	108	4	52	177
Abraham	2005	Case–control	Germany	European	Breast	571/646	12	138	421	16	173	457
McGlynn	2005	Case–control	United States	North American	Colorectal	662/650	13	164	485	15	146	489
Robinson	2005	Case–control	United Kingdom	European	Colorectal	327/322	8	83	236	8	73	241
Shi	2005	Case–control	China	Asian	Hepatocellular	56/60	2	4	50	1	3	56
Gunel‐Ozcan	2006	Case–control	Turkey	Asian	Breast	88/100	0	39	49	1	26	73
Syrjakoski	2006	Cohort	Finland	European	Prostatic	843/480	17	177	649	7	88	385
Syrjakoski	2006	Cohort	Finland	European	Breast	116/480	9	26	89	7	88	385
Cardoso	2006	Case–control	Portugal	European	Cervical	185/135	6	43	136	6	46	85
Kondrashova	2006	Case–control	Russica	European	Breast	99/260	2	30	67	5	75	180
Yonal	2007	Case–control	Turkey	Asian	Hepatocellular	19/251	2	6	11	4	61	186
Hucl	2007	Case–control	Germany	European	Pancreatic	158/549	3	46	109	8	144	397
Ropero	2007	Case–control	Spain	European	Hepatocellular	196/181	9	85	102	5	52	124
Ezzikouri	2008	Case–control	France	European	Hepatocellular	96/226	3	34	59	2	60	160
Nahon	2008	Cohort	France	European	Hepatocellular	103/198	0	28	75	0	49	149
Shi	2009	Case–control	Australia	Oceanican	Colorectal	78/2614	1	18	59	63	732	1819
Shi	2009	Case–control	Australia	Oceanican	Colorectal	70/1605	4	15	51	40	402	1163
Batschauer	2011	Case–control	Brazil	South American	Breast	68/85	6	13	49	3	25	57
Gannon	2011	Cohort	Canada	North American	Ovarian	354/80	8	92	254	3	17	60
Gannon	2011	Cohort	Canada	North American	Endometrial	111/80	4	36	71	3	17	60
Ekblom	2012	Cohort	Sweden	European	Colorectal	218/414	5	42	171	13	96	305
Agudo	2013	Case–control	Spain	European	Gastric	323/1158	11	82	230	23	249	885
Rodriguez‐Lopez	2013	Case–control	Spain	European	AL	59/179	1	9	49	5	60	114
Total						6193/14,024						

H indicates H63D mutant and W indicates wild‐type respectively. AL indicates acute leukaemia.

### Meta‐analysis results

#### C282Y

**Figure 2:**
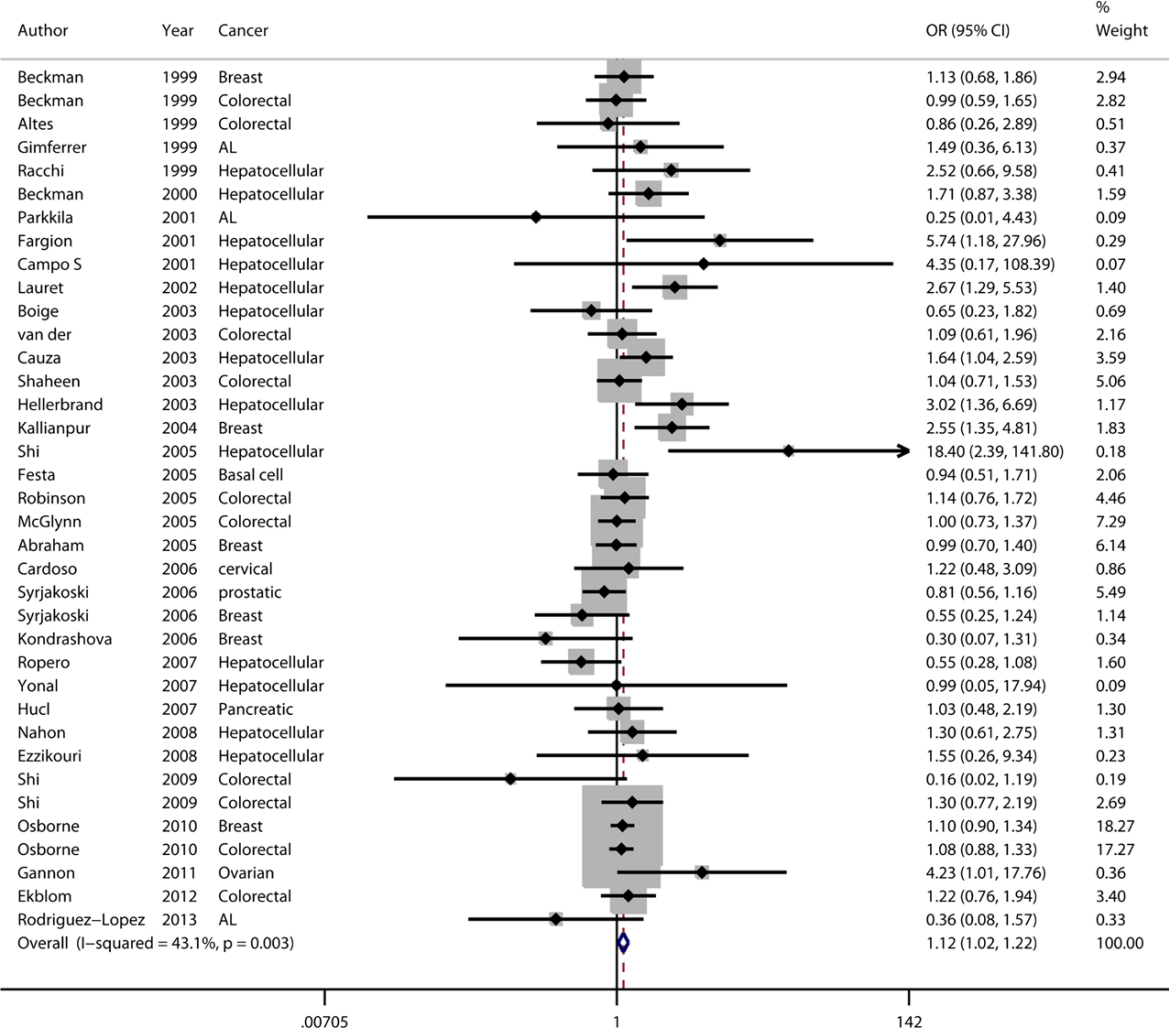
Forest plot (fixed‐effects model) showed C282Y was associated with increased cancer risk in an allele model. Each study is shown by the point estimate of the OR (the size of the square is proportional to the weight of each study) and 95% CI for the OR (extending lines).

The principal findings for C282Y came from 37 data sets from 33 studies, comprising 7487 cases and 59,324 controls (Table 1). Six studies concerned breast cancer 8, 33, 34, 36, 41, 51, nine colorectal cancer 7, 15, 16, 33, 36, 40, 45, 46, 48, thirteen hepatocellular carcinoma 13, 14, 20, 21, 22, 23, 38, 39, 43, 49, 50, 52, 53 and eight studies included six other types of cancer 8, 10, 12, 35, 37, 42, 44, 47 including basal cell carcinoma, cervical cancer, prostatic carcinoma, pancreatic carcinoma, acute leukaemia and ovarian carcinoma. Twenty‐seven studies were European 7, 8, 12, 13, 14, 15, 20, 21, 33, 35, 37, 38, 41, 42, 43, 44, 45, 46, 47, 48, 49, 50, 51, 52, 53, three Oceanian 36, 39, 45, four North American 10, 16, 34, 40 and two Asian 22, 23. Overall, a significantly elevated cancer risk was found according to a recessive genetic model 57 (OR: 1.991, 95% CI: 1.448–2.737) and an allele model 53 (OR: 1.116, 95% CI: 1.024–1.217) (Fig. 2), whereas no statistically significant difference was found in a dominant model 57 (OR: 1.088, 95% CI: 0.992–1.193). Moderate heterogeneity was detected in the dominant model (*P*_h_ = 0.004, *I* ^2^ = 42.3%) and the allele model (*P*_h_ = 0.003, *I* ^2^ = 43.1%), but there was zero heterogeneity in the recessive model (*P*_h_ = 0.632, *I* ^2^ = 0.0%).

On subgroup analysis stratified by cancer type (Table 3), statistically significantly elevated cancer risk was detected in a recessive model for breast cancer (OR: 2.143, 95% CI: 1.24–3.697), hepatocellular carcinoma (OR: 3.642, 95% CI: 1.454–9.122) and colorectal carcinoma (OR: 1.692, 95% CI: 1.041–2.750). The other cancer types showed no significantly increased risk. On subgroup analysis stratified by territory (Table 3), significantly increased risk of cancer was demonstrated in the Oceanian study population in a recessive model (OR: 2.558, 95% CI: 1.657–3.949), in the Asian population in an allele model (OR: 6.975, 95% CI: 1.315–36.999) with significant heterogeneity and in the Asian population in the dominant model (OR: 5.622, 95% CI: 1.014–31.178). No increased cancer risk was found in either European or North American study populations in any genetic model. Heterogeneity was not observed or was slight in all studies, except in an Asian population using an allele model (*P*_h_ = 0.106, *I* ^2^ = 61.7%).

**Table 3:** Pooled analysis of association of C282Y and cancer risk

	Case/control	Dominant model			Recessive model			Allele model		
		(CC+CW) *versus* WW			CC *versus* (CW+WW)			C *versus* W		
		OR	*P*_h_	*I* ^2^	OR	*P*_h_	*I* ^2^	OR	*P*_h_	*I* ^2^
Total	7487/59,324	1.088 (0.992–1.193)	0.004	42.30%	1.991 (1.448–2.737)	0.811	0.00%	1.116 (1.024–1.217)	0.003	43.10%
Cancer type										
Breast	1652/18,211	1.046 (0.884–1.236)	0.031	59.40%	2.143 (1.242–3.697)	0.673	0.00%	1.091 (0.934–1.274)	0.025	61.20%
Colorectal	2865/36,263	1.062 (0.927–1.216)	0.77	0.00%	1.692 (1.041–2.750)	0.523	0.00%	1.073 (0.946–1.219)	0.852	0.00%
Hepatocellular	1152/3131	1.574 (1.217–2.036)	0.016	51.40%	3.642 (1.454–9.122)	0.568	0.00%	1.608 (1.263–2.049)	0.01	54.10%
Others	1818/1719	0.874 (0.662–1.152	0.252	22.30%	1.546 (0.593–4.031)	0.724	0.00%	0.920 (0.709–1.194)	0.324	13.60%
Territory										
European	4376/8758	1.057 (0.921–1.213)	0.01	42.90%	1.255 (0.702–2.244)	0.831	0.00%	1.059 (0.929–1.207)	0.026	37.70%
Oceanican	1531/48,563	1.083 (0.937–1.251)	0.46	0.00%	2.558 (1.657–3.949)	0.795	0.00%	1.142 (1.000–1.305)	0.373	4.00%
Asian	75/311	5.622 (1.014–31.178)	0.261	20.90%	6.647 (0.807–54.756)	0.402	0.00%	6.975 (1.315–36.999)	0.106	61.70%
North American	1505/1692	1.166 (0.917–1.482)	0.029	66.80%	1.682 (0.721–3.923)	0.572	0.00%	1.183 (0.944–1.482)	0.017	70.40%
Begg		*P* = 0.367			*P* = 0.216			*P* = 0.425		
Egger		*P* = 0.217			*P* = 0.100			*P* = 0.334		

*P*_h_: test for heterogeneity, OR: odds ratio, CI: confidence interval.

*I* ^2^: the percentage of total variation across studies that is a result of heterogeneity rather than chance.

C indicates C282Y mutant and W indicates wild‐type respectively.

#### H63D

**Figure 3:**
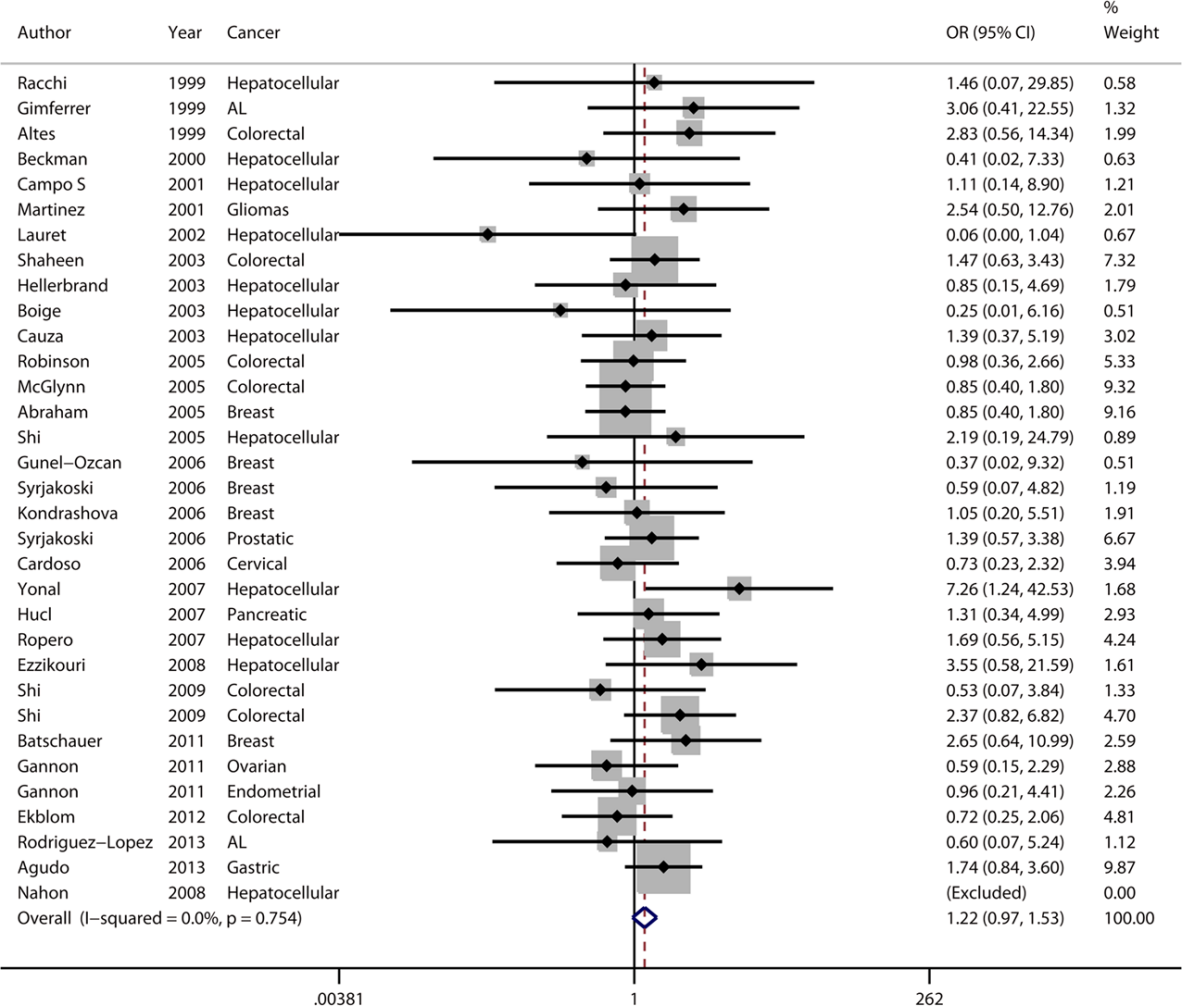
Forest plot (fixed‐effects model) indicated H63D was not associated with increased cancer risk in a recessive model. Each study is shown by the point estimate of the OR combined with 95% CI for the OR. % weight represents the weight of each study.

The results for H63D are comprised of 33 data sets extracted from 30 studies with 6193 cases and 14,024 controls (listed in Table 2). Twelve studies were concerned with hepatocellular carcinoma 13, 14, 20, 22, 23, 38, 39, 43, 45, 49, 50, 52, 53, two with acute leukaemia 12, 47, seven with colorectal cancer 7, 16, 40, 45, 46, 48, five with breast cancer 6, 8, 41, 51, 55 and seven with other neoplasms including glioma 54, prostatic cancer 8, cervical cancer 42, pancreatic cancer 44, ovarian cancer 10, endometrial cancer 10 and gastric carcinoma 9. Twenty‐two studies were European 7, 8, 9, 12, 13, 14, 20, 38, 41, 42, 43, 44, 46, 47, 48, 49, 50, 51, 52, 53, 54, four were North American 10, 16, 40, three were Oceanian 39, 45, three were Asian 6, 22, 23 and one was South American 55. Overall, unlike C282Y, no significant increase in cancer risk was found in any genetic model (Table 4). No heterogeneity (*P*_h_ = 0.754, *I* ^2^ = 0.0%) was found in the recessive model (Fig. 3); the other two models showed significant heterogeneity (dominant – *P*_h_ = 0.002, *I* ^2^ = 46.7%; allele – *P*_h_ = 0.002, *I* ^2^ = 47.2%).

**Table 4:** Pooled analysis of association of H63D and cancer risk

	Case/Control	Dominant model			Recessive model			Allele model		
		(HH+HW) *versus* WW			HH *versus* (HW+WW)			H *versus* W		
		OR	*P*_h_	*I* ^2^	OR	*P*_h_	*I* ^2^	OR	*P*_h_	*I* ^2^
Total	6193/14,024	1.107 (1.025–1.196)	0.002	46.70%	1.215 (0.966–1.528)	0.754	0.00%	1.095 (1.023–1.172)	0.002	47.20%
Cancer type										
Breast	942/1571	1.014 (0.841–1.221)	0.072	53.50%	0.996 (0.555–1.788)	0.629	0.00%	1.010 (0.857–1.191)	0.2	33.20%
Colorectal	1940/6538	1.065 (0.929–1.221)	0.339	11.90%	1.152 (0.781–1.699)	0.496	0.00%	1.064 (0.942–1.202)	0.252	23.20%
Hepatocellular	1068/3003	1.169 (0.988–1.383)	0.051	43.90%	1.447 (0.828–2.529)	0.321	12.90%	1.126 (0.971–1.306)	0.017	52.20%
AL	95/285	0.681 (0.395–1.175)	0.013	83.80%	1.447 (0.333–6.289)	0.279	14.70%	0.785 (0.486–1.268)	0.01	84.80%
Others	2148/2627	1.212 (1.048–1.402)	0.048	52.70%	1.278 (0.844–1.934)	0.719	0.00%	1.191 (1.047–1.355)	0.053	51.70%
Territory										
European	4050/6995	1.089 (0.992–1.195)	0.001	55.70%	1.162 (0.872–1.549)	0.783	0.00%	1.074 (0.989–1.167)	0.001	57.10%
Oceanican	310/4890	0.907 (0.685–1.200)	0.654	0.00%	1.590 (0.742–3.405)	0.411	0.00%	0.960 (0.748–1.232)	0.464	0.00%
Asian	163/411	2.066 (1.280–3.334)	0.946	0.00%	3.147 (0.853–11.612)	0.268	24.00%	1.880 (1.248–2.832)	0.868	0.00%
North American	1602/1643	1.187 (1.001–1.408)	0.683	0.00%	0.986 (0.603–1.611)	0.669	0.00%	1.147 (0.984–1.336)	0.697	0.00%
South American	68/85	0.789 (0.393–1.584)			2.645 (0.636–10.994)			1.010 (0.564–1.809)		
Begg		*P* = 0.963			*P* = 0.466			*P* = 0.963		
Egger		*P* = 0.987			*P* = 0.526			*P* = 0.995		

*P*_h_: test for heterogeneity, OR: odds ratio, CI: confidence interval.

*I* ^2^: the percentage of total variation across studies that is a result of heterogeneity rather than chance.

H indicates H63D mutant and W indicates wild‐type respectively.

Subgroup meta‐analysis was performed according to cancer type and territory. For cancer type, elevated cancer risk was detected in a dominant model for ‘others’, with moderate heterogeneity (*P*_h_ = 0.048, *I* ^2^ = 52.7%). Given that ‘others’ included several types of cancer and that heterogeneity was significant, this result should be viewed with caution. No significantly elevated cancer risk was detected in any other genetic model, suggesting that H63D is not associated with these types of cancer. For territory, increased cancer risk was found in the Asian study population in a dominant model (OR: 2.066, 95% CI: 1.280–3.334, *P*_h_ = 0.946) and an allele model (OR: 1.880, 95% CI: 1.248–2.832, *P*_h_ = 0.868), both with no heterogeneity (*I* ^2^ = 0.0%). In the European, North American, Oceanian and South American populations, no significantly elevated cancer risk was detected in any genetic model.

### Publication bias and sensitivity analysis

**Figure 4:**
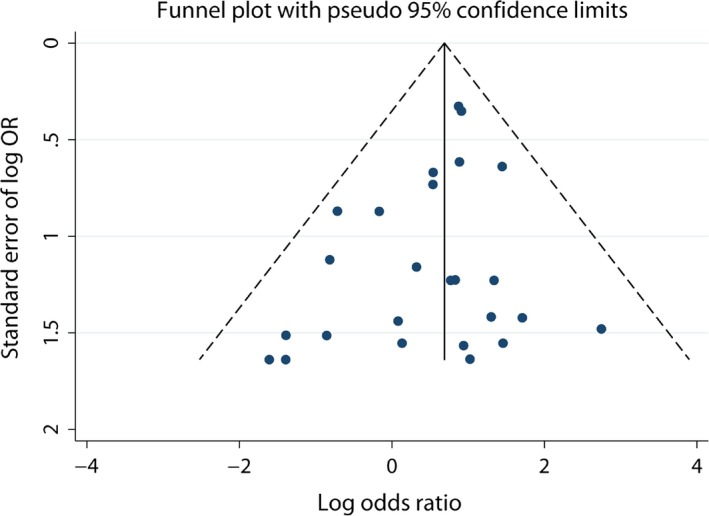
Funnel plot illustrating publication bias (recessive model of C282Y polymorphism).

For C282Y, funnel plots and Begg's and Egger's test were performed to analyse for publication bias in all three genetic models. The shapes of the funnel plots (Fig. 4) appeared symmetrical, indicating no statistically significantly publication bias for the association between C282Y and risk of cancer. This was in agreement with the results from Begg's and Egger's tests (Table 3). Similarly, there was no evidence of publication bias for H63D (Table 4). All of these results indicate that the findings of our study were robust.

**Figure 5:**
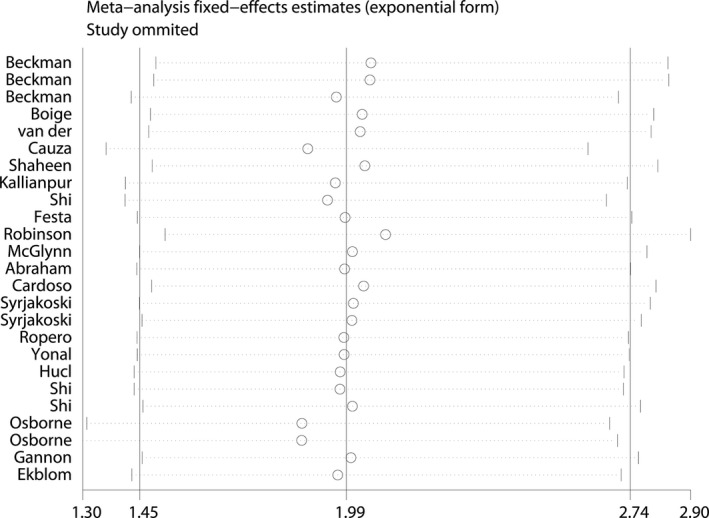
Analysis of the influence of summary odds ratio coefficients on the association between C282Y mutation and cancer risk in the recessive model.

Sensitivity analysis 58 was conducted to determine the publication bias and influence of each study on the pooled OR by sequentially omitting individual studies from the analysis. The series of pooled ORs with 95% CIs lies not far from the midline for the C282Y mutation, which means that the statistical findings were not materially altered by the elimination of any study in the recessive model (Fig. 5). Thus, the possible positive association between C282Y and cancer risk was stable, especially for breast cancer, colorectal cancer and hepatocellular carcinoma.

Similar results were achieved in the sensitivity analysis for H63D mutation, confirming the stability of our findings for H63D.

### Cumulative analysis

**Figure 6:**
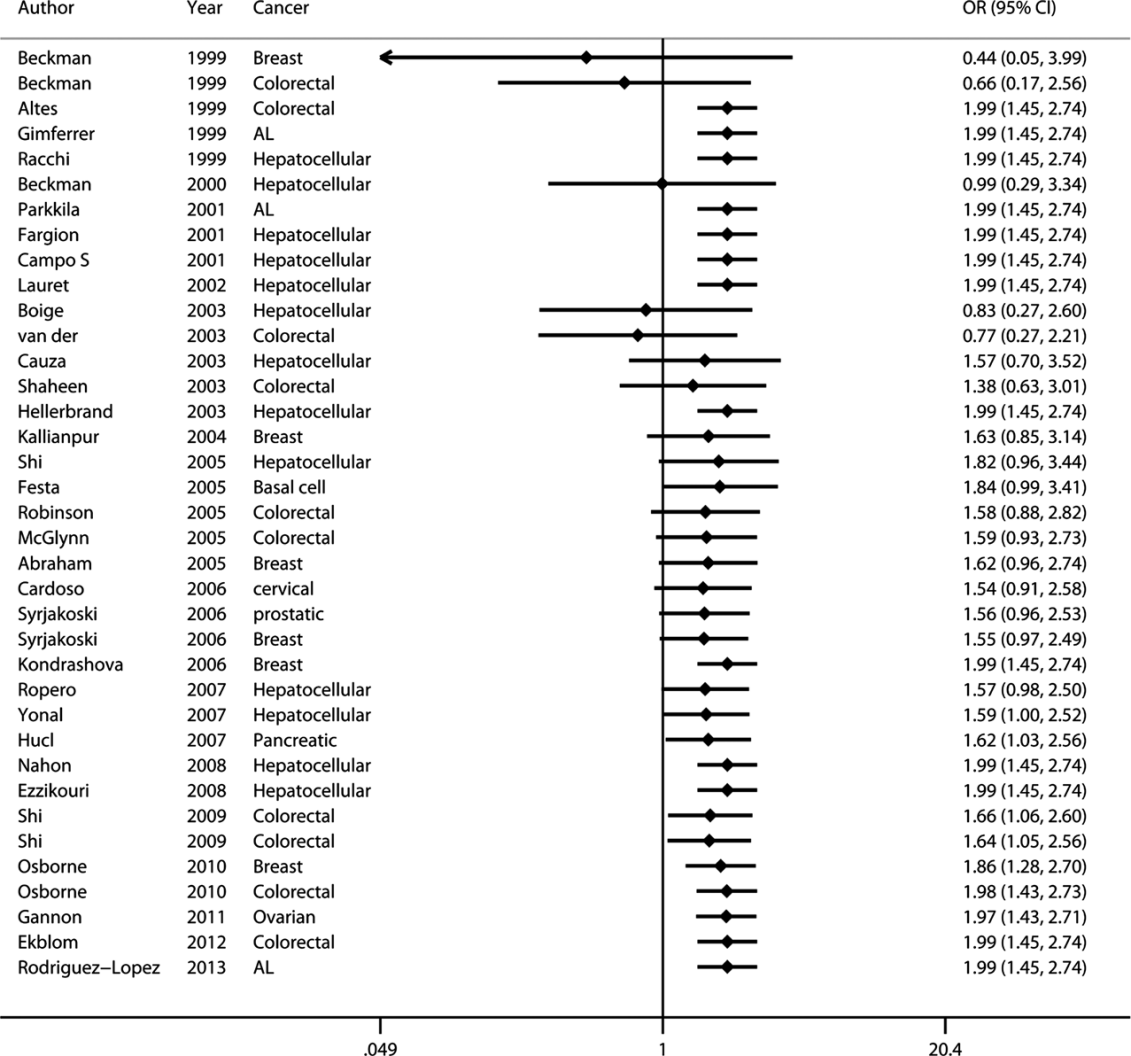
Forest plots for cumulative meta‐analysis of the association between C282Y and cancer risk in the recessive model (year of publication).

Cumulative meta‐analysis 29 was performed by sorting studies by chronological order and sample size. This allows the stability of the research findings over time to be explored. As shown in Figure 6, there is a tendency towards a positive association between C282Y and cancer risk with time. Simultaneously, 95% CIs became narrower, indicating improved precision and accuracy. Increasing sample sizes also narrowed the 95% CIs; the implications being similar.

## Discussion

In this compound study, we performed a meta‐analysis of the association between mutations of the HFE gene and risk of cancer including 36 eligible case–control or cohort studies. Thirty‐three studies concerned the C282Y mutation, with 7487 cases and 59,324 controls. C282Y was found to increase the risk of cancer twofold in the recessive model and 1.1‐fold in the allele mode. On stratified analysis by cancer type, a statistically significant increase was found for breast cancer, colorectal cancer and hepatocellular carcinoma in the recessive model, in accordance with the studies of Jin *et al*. 4, Chen *et al*. 24 and Liu *et al*. 25. These results suggest that the C282Y/C282Y genotype is associated with a twofold elevated risk for breast cancer, a 1.7‐fold elevated risk of colorectal, and a 3.6‐fold increased risk of hepatocellular cancer. There is insufficient evidence to conclude that it is a risk factor for other types of cancer. Subgroup analysis stratified by territory showed that the C282Y mutation was associated with a 2.6‐fold increased risk of cancer in Oceanian populations in a recessive model and by 6.9‐fold in Asian populations in an allele model. These findings suggest that the living environment, genetic background and dietary habits are candidate factors that influence the risk of cancer because of HFE mutations. This is the most comprehensive study reported to date, evaluating the association between HFE genotype and overall cancer risk, with stratification based on territory.

H63D, another missense mutation of the *HFE* gene, was investigated in thirty studies with 6193 cases and 14,024 controls. We found that H63D did not increase the overall cancer risk or the risk of particular types of cancer on subgroup analysis, with ORs only slightly over 1 in all genetic models. However, the result of ‘others’ showed H63D increased cancer risk 1.2‐fold in both dominant and allele models. Given that ‘others’ included several types of cancer, and that the heterogeneity in both model was moderate, we advise that these findings should be viewed with caution. Our results indicated that H63D is a weak or irrelevant factor in the development of cancer. However, in the Asian study population, H63D was found to be related to elevated cancer risk in both a dominant by twofold and an allele model by 1.9‐fold, suggesting a possible role for genetic background, diet and lifestyle, and environmental conditions.

Generally, it could be concluded from our study that the C282Y mutation, especially the C82Y/C282Y genotype, is a risk factor for cancer. The association between C282Y and breast, colorectal and hepatocellular carcinoma was statistically significant. However, H63D was not a distinct risk factor or only a weak one. It is well known that HFE is an atypical major histocompatibility complex class I molecule, affecting iron load and immune function through its interaction with β2 microglobulin (β2 m) and the TfRs (TfR1 and TfR2) 59, 60. Generally, normal HFE associates with β2 m, transits to the membrane, and binds with TfRs. When combining with TfR1, HFE competes with transferrin to limit the rate of iron uptake, promoting a homoeostatic level of iron load. However, when forming a complex with TfR2, it stimulates the secretion of Hepcidin, thus suppressing the iron export protein ferroportin and promoting cells to retain iron intracellularly. All these finding indicated that HFE plays vital role in iron homoeostasis regulation 61. Expectedly, mutations in HFE cause the disruption of HFE function, leading to iron overload. Specifically, C282Y polymorphism cannot interact with β2 m, preventing its surface translocation and variant H63D translocates to the cell surface but fails to participate in the interactions with the TfR1, which might promote the interaction with TfR2 in hepatocytes, causing a systemic increase in hepcidin and suppression of ferroportin 59, 62.

The mechanism of the damage caused by excess iron might be related to the creation of free radicals during the Fenton reaction, leading to the formation of reactive oxygen species (ROSs). It is known that ROSs can cause lipid peroxidation, protein modification, and DNA and RNA mutations, thus resulting in dysregulation of normal cell functioning, pathological states and cell death 63, 64. Specifically, intracellular iron overload leads to cell cycle arrest at the G1/S stage by affecting the expression of certain cyclins and protein kinases. Reactive oxygen species can react with DNA, causing damage, mutation, oncogene activation or inactivation of cancer suppressor genes. In addition, hydroxyl radicals may cause apoptosis 65 because of their effects on mitochondrial and lysosomal membranes.

As suggested by the American Association for the Study of Liver Diseases, phlebotomy is the principle treatment for hereditary haemochromatosis, being an effective method for maintaining serum ferritin levels. Thus, a number of the cases included in our study had probably undergone phlebotomy, which would have reduced their serum ferritin levels and might have reduced their susceptibility to cancer. This may have affected the results of our study.

Our study has limitations. First, our meta‐analysis was based on unadjusted related data, and any confounding factors could not be controlled for because most of the included studies did not provide any relevant data. Second, the sample sizes of several of the studies might not have been large enough to detect any possible risks associated with the HFE mutations. This is most likely to have applied to the results concerning Oceanian and Asian populations. Third, because cancer is a complex disease with a multifactorial aetiology, gene–gene and gene–environment interactions should be evaluated; however, we did not address this in our study. Last, most of the studies included in our meta‐analysis were concerned with breast cancer, colorectal cancer or hepatocellular carcinoma; those concerning several other types of cancer were simply combined together as ‘others’. As a consequence, our findings with these studies might not be precise. We hope to address this in future studies.

In conclusion, this is a comprehensive meta‐analysis concerning HFE gene mutation (C282Y and H63D) and overall cancer risk. The C282Y mutation was associated with increased overall cancer susceptibility, especially for hepatocellular carcinoma, breast cancer and colorectal cancer, whereas the H63D mutation produced non‐significant results for these three types of cancer. The effect of territory on the association between HFE mutation and cancer could be a factor in susceptibility. Further well‐designed epidemiological studies of cancer types and territory and large‐scale studies concerning gene–gene or gene–environment interactions should be conducted to clarify the association. The molecular mechanism of how C282Y increases cancer risk also merits further study, to aid understanding of the role of HFE gene mutation in carcinogenesis.

## Conflicts of interest

The authors disclose no potential conflicts of interest.

## Supporting information


**Table S1** Summary odds ratios for C282Y.
**Table S2** Summary odds ratios for H63D.
