# Supplementary material for: The risk of new‐onset cancer associated with HFE C282Y and H63D mutations: evidence from 87,028 participants
Source: J Cell Mol Med. 2016 Feb 19;20(7):1219–33. doi: 10.1111/jcmm.12764 (PMC4929296; doi:10.1111/jcmm.12764)
Supplement: Supplementary file 1 — Table S1 Summary odds ratios for C282Y. Table S2 Summary odds ratios for H63D. [file JCMM-20-1219-s001.doc]

Supplementary table 1. Summary odds ratios for C282Y.

| Comparision | OR | 95% CI | | *P*_OR_ | Bon |
| --- | --- | --- | --- | --- | --- |
| C vs W | 1.116 | 1.024 | 1.217 | 0.012 | 0.024 |
| CC vs CW | 1.887 | 1.348 | 2.641 | 0.000 | 0.000 |
| CW vs WW | 1.055 | 0.959 | 1.160 | 0.274 | 0.548 |
| CC vs WW | 2.016 | 1.466 | 2.773 | 0.000 | 0.000 |
| CC vs CW+WW | 1.991 | 1.448 | 2.737 | 0.000 | 0.000 |
| CC +CW vs WW | 1.088 | 0.992 | 1.193 | 0.073 | 0.146 |

Bon: *P vaule in* Bonferroni testing.

OR: odds ratio, CI: confidence interval,

C indicates C282Y mutant and W indicates wild type, respectively

Supplementary table 2. Summary odds ratios for H63D.

| Comparision | OR | 95% CI | | *P*_OR_ | Bon |
| --- | --- | --- | --- | --- | --- |
| H vs W | 1.095 | 1.023 | 1.172 | 0.009 | 0.018 |
| HH vs HW | 1.135 | 0.894 | 1.440 | 0.299 | 0.598 |
| HW vs WW | 1.105 | 1.020 | 1.197 | 0.014 | 0.028 |
| HH vs WW | 1.241 | 0.985 | 1.562 | 0.066 | 0.132 |
| HH vs HW+WW | 1.215 | 0.966 | 1.528 | 0.096 | 0.192 |
| HH +HW vs WW | 1.107 | 1.025 | 1.196 | 0.010 | 0.020 |

Bon: *P vaule in* Bonferroni testing.

OR: odds ratio, CI: confidence interval,

H indicates H63D mutant and W indicates wild type, respectively
